# The Rectum: Its Diseases and Developmental Defects

**Published:** 1909-06

**Authors:** 


**The Rectum: its diseases and developmental defects.** By Sir CHARLES B. BALL, M.Ch. Pp. xiv, 332. London: Oxford Medical Publications. 1908.

We are greatly pleased with this book and the excellence of the illustrations. They are as good as those in Kelley's works on gynæcology and diseases of the vermiform appendix. Photographs were taken with a stereoscopic camera, and drawings made from them, so as to avoid the flat appearance of an ordinary photograph. The result is excellent. We think the value of such a work has been greatly increased by these illustrations, as no amount of description will sometimes convey to the mind what a glance at a good picture will do.

Sir Charles Ball tells us that we shall not find an exhaustive reference to the works of other surgeons, and this certainly is so, though the French surgeons seem to be referred to fairly often. No doubt such reference is desirable, but on the other hand a book must be brought within reasonable limits, and Sir Charles Ball's experience is well worth reading apart from that of others. The work is not really a very lengthy one, though the large size of the volume might suggest that it was. We have the luxury of very large type.

It opens with a full description of the anatomy of the rectum, the value of which is much increased by the excellence of the illustrations already referred to. Ball describes a new method of naming the lower bowel—introduced by Jonnesco—so that the rectum is said to begin at the third sacral vertebræ, and the bowel above is called the pelvic colon. But he does not adopt the nomenclature in his later description of the rectum, and this is a little confusing.

We are very glad to see that Sir Charles Ball is opposed to the practice—advocated by some surgeons—of making a deep dissection from the perineum to find the rectum in cases of imperforate anus, when there is no indication that the rectum is near the perineum. We have for many years felt that the best thing to do was not to follow this advice, but rather to do what Sir Charles Ball recommends—an iliac colotomy. What value can a long contracting track—practically a sinus—running from the perineum some inches up to the opened rectum be? If we have to dissect deeply to find the blind end of the rectum, there is little chance of bringing it down and uniting it to the skin, without such tension that it would almost certainly break away again; and if it did unite, what sphincteric control could there be? The external sphincter could not possibly be made to surround the opened bowel.

When we remember what good use has already been made of the sigmoidoscope in diagnosing growths high up in the rectum and sigmoid, we think Sir Charles Ball hardly gives due importance to such methods of examination, and we are rather disappointed he has not used the method more, and devoted a chapter to its use. Touch is, no doubt, as he says, a most valuable method of examination, but even a bimanual examination under an anæsthetic may fail to reveal a high growth which a proctoscope or sigmoidoscope may disclose without the need for an anæsthetic at all.

The author's further experience has convinced him of the importance of recognising the torn down anal valve—the "sentinel pile"—as a cause of fissure of the anus, and that the condition is best treated by simply removing the "pile," and that division of the fibres of the sphincter is unnecessary. We must say that in many fissures we have had to treat, although we have looked for the "sentinel pile"—since we read Sir Charles Ball's paper on the subject some years ago—we have not found it.

In describing the treatment of piles, the author gives information only about his own method of operating, and a brief reference to Whitehead's method. His own method is doubtless an excellent one—it is a form of ligature—but the method of Mitchell, so frequently used now, might, we think, have received some recognition. He does not favour Whitehead's operation, because of the almost inevitable retraction of the sutured mucous membrane. With regard to the impossibility of preventing this in many cases, we quite agree with him, for we have tried a special method of deep suture without success. He writes in a very encouraging way about the results of his little operation for pruritus ani. Even though sensibility return to the area of skin dissected up, the pruritus does not return.

Sir Charles Ball does not give us details and statistics of his cases of cancer of the rectum, as Mr. Harrison Cripps did in his recent work on the subject and in his book on rectal diseases; but by means of his excellent plates and a clear description he gives us useful practical details of operative treatment, and a summary of his experience of its indication and result. He truly says it is no use preserving the external sphincter in the perineal operation if we then cut its nerve supply by dividing the levator ani at its connection with the rectum—as is done in Quenu's operation—and thus dividing the inferior hæmorrhoidal nerve on each side. He has devised an operation for preserving these nerves and the sphincter, which he fully describes; but the question it seems to us is whether, if the growth was situated high enough above the anus for the operation, the anal segment and external sphincter need be disturbed at all—whether a coccygeal or sacral operation might not be done instead.

We have already expressed our thorough agreement with Sir Charles Ball in his treatment of imperforate anus. There is another matter with regard to which we are delighted to see his opinion, and that is with regard to colotomy in cancer of the rectum too advanced for excision. Why, as he says, should colotomy be done by some surgeons as a routine thing in such cases ? The patient may never get obstruction at all. If the lumen is very small and there are already signs of oncoming obstruction, that is another matter. But to do colotomy to secure " physiological rest " is, as Sir Charles Ball points out, to operate with very little reason.

The book is a most valuable one; but essentially a practical guide and not an exhaustive treatise, but no doubt for that reason all the more welcome to many who find life all too short for the many demands of study and work.

